# drGAT: Attention-Guided Gene Assessment of Drug Response Utilizing a Drug-Cell-Gene Heterogeneous Network

**Published:** 2024-05-14

**Authors:** Yoshitaka Inoue, Hunmin Lee, Tianfan Fu, Augustin Luna

**Affiliations:** 1 Department of Computer Science and Engineering, University of Minnesota; 2 Computational Biology Branch, National Library of Medicine; 3 Computer Science Department, Rensselaer Polytechnic Institute; 4 Developmental Therapeutics Branch, National Cancer Institute

## Abstract

Drug development is a lengthy process with a high failure rate. Increasingly, machine learning is utilized to facilitate the drug development processes. These models aim to enhance our understanding of drug characteristics, including their activity in biological contexts. However, a major challenge in drug response (DR) prediction is model interpretability as it aids in the validation of findings. This is important in biomedicine, where models need to be understandable in comparison with established knowledge of drug interactions with proteins. drGAT, a graph deep learning model, leverages a heterogeneous graph composed of relationships between proteins, cell lines, and drugs. drGAT is designed with two objectives: DR prediction as a binary sensitivity prediction and elucidation of drug mechanism from attention coefficients. drGAT has demonstrated superior performance over existing models, achieving 78% accuracy (and precision), and 76% F1 score for 269 DNA-damaging compounds of the NCI60 drug response dataset. To assess the model’s interpretability, we conducted a review of drug-gene co-occurrences in Pubmed abstracts in comparison to the top 5 genes with the highest attention coefficients for each drug. We also examined whether known relationships were retained in the model by inspecting the neighborhoods of topoisomerase-related drugs. For example, our model retained TOP1 as a highly weighted predictive feature for irinotecan and topotecan, in addition to other genes that could potentially be regulators of the drugs. Our method can be used to accurately predict sensitivity to drugs and may be useful in the identification of biomarkers relating to the treatment of cancer patients.

## 1 Introduction

Drug discovery is an expensive and lengthy process with many obstacles Azuaje (2017); Kuenzi et al. (2020). The difficulty of this process arises from the efforts needed to ensure a compound is both safe and effective. Sensitivity to a drug involves the mechanism of the drug compound and a complex interplay of various factors internal and external to a cell. These factors include the cellular context (or state) that is determined by the repertoire of transcripts and proteins, alterations to this repertoire due to disease, and the interactions between these components Chang et al. (2019). Biomarkers are crucial in drug development to understand the best use of a compound and aid our understanding of the disease biology Garnett et al. (2012); Lu et al. (2022); Chen et al. (2021). Machine learning (ML) has emerged as an approach to understanding the role of particular genes in drug response. This makes a huge impact on the efficiency and success rates of developing new drugs Leung et al. (2015). The utilization of ML in this domain leverages large amounts of biological and chemical data, helping to identify drug candidates and predict their efficacy efficiently. When it comes to understanding biological phenomena, ML algorithms, especially deep learning (DL) models, have demonstrated their capability to predict the complex patterns and relationships within biological data Cao et al. (2018); Mahmud et al. (2020).

However, despite the advances in using machine learning for drug discovery, a critical difficulty remains; which is the “black box” nature of the methods. While DL models have made huge strides in identifying patterns and predicting outcomes within several areas, their internal decision-making process is unclear, leading to concerns about their trustworthiness and reliability.

Interpretability in machine learning has received significant recent consideration Lipton (2018); Gilpin et al. (2018); Kengkanna and Ohue (2023) including in the area of drug discovery Fu et al. (2022, 2021). The attention mechanism, introduced in the Transformer architecture Vaswani et al. (2017), is being widely used due to its capacity to include “attention” as trainable parameters to optimize how much attention the model assigns to individual components, such as a word in a sentence (*i.e.*, a linear sequence of data); these attention parameters can be used to interpret the model’s output. While words in a sentence express a single coherent idea, regulatory networks biology are more dependent on interconnections between components; this makes graph data structures a useful representation in biology. Graph Neural Networks (GNNs) have been developed for handling this type of data. GNNs that make use of the attention mechanism are known as Graph Attention Network (GAT) Veličković et al. (2017). Thus, GAT leverages the attention mechanism to generate attention weights, indicating the weight from one node to others assigned to each component. By utilizing GAT, we can offer insights into the importance of genes and enhance the interpretability of drug response models by analyzing neighborhood relationships.

We propose a novel interpretable deep learning method called “drGAT”, which leverages GAT to process a large-scale heterogeneous network. Our model inputs a heterogeneous graph composed of drug compounds, cell lines, and genes. This heterogeneous graph has been constructed using integrated drug screening and molecular profiling data from the NCI60 cell line pharmacogenomics dataset (drug screening data collected on 60 cancer cell lines) taken from CellMinerCDB Luna et al. (2021) via rcellminer Luna et al. (2016). The GAT layer is employed to learn embedding representations from the interconnected structure within this heterogeneous network. This approach gives us knowledge of the relative significance of individual components Yuan et al. (2021, 2022) and their contributions to the overall model.

We utilize drGAT for two tasks: i) drug-cell association prediction and ii) interpretability of the individual gene importance to prediction. We make use of multi-task learning, whereby multiple learning tasks are solved simultaneously to benefit from the shared patterns and distinctions among these tasks. Towards this aim, this study focuses on drug compounds with a DNA-damaging mechanism of action (269 drugs) available in the NCI60 of which there are more than in other comparable datasets. Regarding drug response prediction, drGAT has demonstrated superior performance over existing models. Additionally, this model also shows high prediction accuracy on data not present in NCI60 but found within the GDSC dataset.

In addition, the attention coefficients from drGAT are utilized for interpreting the predictor (*e.g.*, gene) importance and relation among components of the heterogeneous network nodes (*i.e.*, drugs, cell lines, and genes). We present an analysis of attention coefficients for the subset of nodes that correspond to genes that propose explanations for the response mechanisms of particular drugs. Moreover, relevant biological processes for analyzed compounds are also described using the attention coefficients and an over-representation analysis.

In summary, our model shows high accuracy for drug response prediction, allowing us to determine the predictor importance using attention coefficients. This interpretability allows users to explore the relationship among drug structures, cell lines, and genes, providing insights for further drug development investigation.

The main contributions include:

1. We create a heterogeneous graph involving drug compounds with DNA damage that includes three entity types: genes (*i.e.,* gene expression), cell line (*i.e.,* drug responses), and drug (*i.e.,* structures) using the NCI60 Shoemaker (2006).
2. We evaluate the drug-target interaction from attention coefficients and systematically compare it with existing scientific literature. We examine the abstracts of journal papers for co-mentions of drug-target relationships from our results.
3. Our results propose drug-cell line sensitivity associations based on the attention coefficients derived from the GAT. These associations were then validated through comparison with independent experimental data.

## 2 Related Works

Drug response prediction remains a challenging research area due to the biological factors involved. A central goal of the work is to identify novel drug-cell line sensitivity associations where a cell line’s sensitivity to a drug compound can be reliably predicted. Machine learning-based approaches have become widely used for this task Chen and Zhang (2021). These methods often use several data types, such as gene expression data Kim et al. (2021) and alteration data, as well as, chemical structures of drug compounds An et al. (2022).

Of such methods, Similarity-Regularized Matrix Factorization (SRMF) Wang et al. (2017), a Heterogeneous Network-based Method for Drug Response Prediction (HNMDRP) Zhang et al. (2018), and a Multi-Omics Data Fusion and Graph Convolution Network (MOFGCN) Peng et al. (2021) all leverage the similarity of drugs and cells, which we also utilize.

SRMF Wang et al. (2017) employs matrix factorization Koren et al. (2009) to reconstruct the association matrix using drug-to-drug and cell line-to-cell line similarities. This reconstruction considers the input of similarity metrics encapsulating the relationship between cell lines and drugs. Specifically, SRMF calculates the extent of similarity amongst various drugs, considering their distinct chemical structures, in tandem with assessing the similarity between cell lines and their respective gene expressions. By these similarity quantifications, SRMF undertakes matrix factorization for drugs and cell lines separately. From both matrices, SRMF reconstructs an association matrix.

HNMDRP Zhang et al. (2018) combines gene expression, drug structures, and protein-protein interaction (PPI) networks to predict drug-cell sensitivity associations. The proposed methodology integrates various data types, including gene expression data for cell similarity determination, chemical structures for drug similarity establishment, and PPI information for calculating the similarity of drug target genes. The associations between drugs and cell lines are predicted using this network.

MOFGCN Peng et al. (2021) utilizes Graph Convolutional Networks (GCNs) Kipf and Welling (2016) to predict drug response from the drug and cell line similarity. The cell line similarity matrix is constructed from gene expression, somatic mutation, and copy number variation data, while the drug similarity matrix is derived using structure fingerprints taken from the PubChem database Bolton et al. (2008). Subsequently, an association matrix was created from both matrices and utilized for an input of GCNs to predict drug-cell binary sensitivity associations.

Here, SRMF, HNMDRP, and MOFGCN make predictions using similarity. However, the methods use embeddings utilizing gene expression data, resulting in a potential loss of interpretability. In contrast, our method drGAT integrates gene expression into similarity and association matrices, enabling the measurement of the influence of genes on each drug effect.

## 3 Methods

### 3.1 Building Input Matrix

In this section, we explain the construction of the heterogeneous graph. The resulting graph network incorporates drug structures, cell line drug response, gene expression data, and their known drug-target interactions and similarities.

#### 3.1.1 Preprocessing

First, we selected a subset of data from NCI60 Shoemaker (2006) via rcellminer Luna et al. (2016). This dataset comprises gene expression, drug response, and drug structure data.

The complete drug response matrix contained 23,191 drugs for 60 cells. From this, we selected 269 drugs with a mechanism of action related to DNA damage Weber and Weber (2015). Specifically, the drug response matrix forms $X_{DR}\in R^{n\times m}$, where n is the number of drugs and m is the number of cells (*i.e.*, n=269, m=60).

The full NCI60 gene expression data includes 23,826 gene transcripts, where we selected 2,383 genes (the top 10% of genes with the greatest standard deviations). The selection of this highly variable gene set should allow better discrimination between different observations in the dataset. We also merged another group of genes (281 genes) that are involved in drug-target interactions. This combination resulted in a unique set of 2,718 genes. Notably, the gene expression data $X_{GE}$ is conveyed within a matrix representation, denoted as $X_{GE}\in R^{m\times l}$, wherein m denotes the number of cells and l signifies the count of genes (*i.e*., m=60, l=2,718).

Subsequently, we collected the drug-target interaction data from DrugBank Wishart et al. (2018). This dataset has 19,017 drug-target interactions (DTI) with 7,756 unique drugs and 4,755 genes. We selected 100 unique National Service Center (NSC) numbers (NSCs represent batches of screening with a particular drug structure) and 403 genes these drugs and genes overlap with the NCI60 pharmacogenomics data. To be the same size as other data, the matrix $X_{DTI}$ is filled by 0 for the unknown DTI. Therefore, $X_{DTI}\in R^{n\times l}$ (*i.e.*, n=269, l=2,718).

In addition, we also created the drug fingerprints representation matrix. SMILES structures are converted to the Morgan fingerprints Morgan (1965) using RDKit Landrum et al. (2016). We create a vector of 2048 length for each drug and concatenate to make a matrix, denoted as $X_{MF}\in R^{n\times o}$, wherein o represents the number of drug features (*i.e.*, m=60, o=2,048).

#### 3.1.2 Feature Matrix

To make a feature matrix, we utilize similarity matrix as input matrixes created from $X_{DR}$, $X_{GE}$, and $X_{MF}$. Preceding the construction of the heterogeneous graph, similarity matrices were individually constructed for cells $\left(S_{c}\right)$, genes $\left(S_{g}\right)$, and drugs $\left(S_{d}\right)$. These matrices capture the element-wise similarity to homogenous entities such as gene-gene, cell-cell, and drug-drug similarity relationships.

The RBF Kernel Burbidge et al. (2001) was employed to create a similarity matrix, defined as follows.

(1) $$S_{ij}=\exp\;\left(-\gamma {∥X_{i}-X_{j}∥}^{2}\right),$$

where S is a similarity matrix, γ denotes a hyperparameter (we utilize $\frac{1}{\text{length}\left(X_{i}\right)}$), i and j are indexes, and X is an input matrix.

From Equation (1) with $X_{DR}$, we obtained the cell similarity matrix $S_{c}$, and the gene similarity matrix $S_{g}$ was built using Equation (1) based on the gene expression data $X_{GE}$. Then, Equation (1) was applied to obtain the similarity matrix to the $X_{MF}$ to create a drug similarity matrix $S_{d}$.

As a result, three similarity matrices were obtained: $S_{c}\in R^{m\times m}$ for cells, $S_{d}\in R^{n\times n}$ for drugs, and $S_{g}\in R^{o\times o}$ for genes. These matrices have different sizes, and therefore, we utilize a linear layer to create a unified matrix. Thus, the final feature matrix is defined as follows:

(2) $$X=\left[\begin{array}{ccc} {\text{Linear}}_{d}\left(S_{d}\right) & 0 & 0\\ 0 & {\text{Linear}}_{c}\left(S_{c}\right) & 0\\ 0 & 0 & {\text{Linear}}_{g}\left(S_{g}\right) \end{array}\right],$$

where each linear layer has a different input size and the same output size. The output size is a hyperparameter.

#### 3.1.3 Adjacency Matrix

For the input matrix, we also create the adjacency matrix from $X_{DR}$, $X_{GE}$, and $X_{DTI}$. The drug-cell association matrix $A_{dc}$ is derived from the drug response matrix, $X_{DR}\in R^{n\times m}$. Values in this matrix have already been normalized to z-scores with an average value of 0 and a standard deviation of 1. Then, we applied the following procedures to make it a graph structure. If the z-score in the drug response data for the NCI60 is greater than 0, we consider that the cell line is sensitive to the drug, and the value is 1. Otherwise, the cell line is resistant, and the value is set to 0.

Subsequently, we establish a cell-gene association matrix, $A_{cg}$, derived from the gene expression dataset $X_{GE}\in R^{m\times l}$. Our initial step includes standardizing the data across rows (cell lines). After this, the cell-gene association matrix $A_{cg}$ is generated from $X_{GE}$ processing similarly to drug response matrix $X_{DR}$.

Lastly, we create the association matrix between drugs and genes from the drug-target interaction matrices $X_{DTI}$. To make a matrix with binary values, datasets with numerical values are binarized with values greater than 0 set to 1; otherwise, they are set to 0. Then, all datasets are merged as the drug-gene association matrix $A_{dg}\in R^{n\times l}$ whereby if an interaction is present in any of the datasets, it will be retained in the final merged association matrix.

Based on the three matrices, a unified feature matrix F is created as the following Equation (3). $F\in R^{\left(n+m+l)\times (n+m+l\right)}$ represents the combined drug, cell, and gene components, where the dimension of F is (4430, 4430).


(3) $$A=\left[\begin{array}{ccc} 0 & A_{dc} & A_{dg}\\ A_{dc}^{⊤} & 0 & A_{cg}\\ A_{dg}^{⊤} & A_{cg}^{⊤} & 0 \end{array}\right].$$

In this process, the adjacency matrix A with non-zero values is represented as 1; otherwise, 0. From these processes, the input matrix X and the adjacency matrix A are utilized for the input of the GNN model.

#### 3.1.4 Masking to Avoid Leakage

For evaluation, the drug response matrix $X_{DR}$ is partitioned into distinct subsets: training, validation, and test data (for precise specifications, refer to Section 4.1). The test dataset incorporates the set $A_{dc}$. Therefore, it is imperative to address the concern of potential data leakage. To counteract this issue, we undertook the masking of association values linked with the test data (20 % of total combinations) by rendering 3,228 combinations to a value of 0, utilizing the subsequent equation:

(4) $${\left(A_{dc}\right)}_{ij}=\left\{\begin{array}{ll} 0, & \text{if}\;(i,j)\;\text{is}\;\text{an}\;\text{index,}\\ {\left(A_{dc}\right)}_{ij}, & \text{otherwise,} \end{array}\right.$$

where i is the index of a drug, and j is the index of the cell for test data.

### 3.2 drGAT Model

This model consists of two GAT layers and a single fully connected layer. Equation (5) illustrates the first block of the GAT layer with feature matrix X and adjacency matrix A. The subsequent steps involve adapting the model’s output to predict drug sensitivity in a binary manner.

(5) $$Z_{1}=\mathrm{Dropout}\;\left(\mathrm{ReLU}\;\left(\mathrm{GraphNorm}\;\left(\mathrm{GAT}\left(X,A\right)\right)\right)\right),$$

where Dropout denotes the dropout layer, ReLU is a ReLU activation function, GraphNorm is a graph normalization layer Cai et al. (2021), and GAT is a Graph Attention Layer.

Here, we utilized Graph Attention Layer (GAT) Veličković et al. (2017) defined as follows:

(6) $$\text{GAT}\left(x_{i}\right)=\alpha_{ii}W_{s}x_{i}+\sum_{j\in 𝒩\left(i\right)}\alpha_{ij}W_{t}x_{j},$$

where $x_{i}$ is an input, α is the attention coefficient matrix, $W_{s}$ and $W_{t}$ are the weight matrix, and 𝒩(i) is some neighborhood of node i in the graph. Here, the attention coefficients $\alpha_{ij}$ are computed as

(7) $$\alpha_{ij}=\frac{exp\;\left(LReLU\;\left(a_{s}^{⊤}W_{s}x_{i}\;+\;a_{t}^{⊤}W_{t}x_{j}\right)\right)}{\sum_{k\in 𝒩(i)}\;\;exp\;\left(LReLU\;\left(a_{s}^{⊤}W_{s}x_{i}\;+\;a_{t}^{⊤}W_{t}x_{k}\right)\right)},$$

where $a_{s}$ and $a_{t}$ are weight vectors, and LReLU describes LeakyReLU. This attention coefficient matrix α is utilized for further interpretability assessment.

From $Z_{1}$, we utilize the same structure and obtain $Z_{2}$ as follows.


(8) $$Z_{2}=\mathrm{Dropout}\;\left(\mathrm{ReLU}\;\left(\mathrm{GraphNorm}\;\left(\mathrm{GAT}\left(Z_{1},A\right)\right)\right)\right).$$

Then, this matrix was transformed to match the drug-cell association matrix by concatenation, and then this matrix serves as the fully connected (FC) layer's input. Equation (9) details the entire computational procedure. Then, the predicted value, $\overset{ˆ}{y}$ is obtained by taking the sigmoid function to the output of an FC layer as follows:

(9) $$\overset{ˆ}{y}=\mathrm{sigmoid}\left(\mathrm{FC}\left(Z_{2d}∥Z_{2c}\right)\right),$$

where the $Z_{2d}$ and $Z_{2c}$ are referred to as the drug's and cell's embedding from the $Z_{2}$, respectively, and ∥ describes concatenation. Then the output y and ground truth $\overset{ˆ}{y}$ are fed into the binary cross entropy loss L as below:

(10) $$L\left(y,\hat{y}\right)=-\frac{1}{N}\sum_{i=1}^{N}\left[y_{i}\;\text{log}\;\left({\hat{y}}_{i}\right)+\left(1-y_{i}\right)\;\text{log}\;\left(1-{\hat{y}}_{i}\right)\right],$$

where N is the number of data. Note that this network utilized Adam Kingma and Ba (2014) for optimization.

## 4 Experiment

### 4.1 Cross Validation

The drug response matrix $X_{DR}$ is utilized as the input to the prediction model, using a split of 60% for training, 20% for validation, and 20% for testing. The drug response matrix contains z-score values that are transformed into a binary association matrix as follows: if the value in the drug response data for the NCI60 is positive, we consider that the cell line is sensitive to the drug and set the value to 1; otherwise, the cell line is resistant and set the value to 0.

### 4.2 Baseline Methods

For comparison, we have selected four recent studies as benchmark models. As discussed in section 2, our work is related to MOFGCN, a current state-of-the-art method that uses a heterogeneous graph from drug response and gene expression. Consequently, MOFGCN is our primary benchmark model for GNN-based methods. Furthermore, we include a deep learning method to predict the drug sensitivity of cancer cell lines, called DeepDSC Li et al. (2019) for the DNN-based methods. DeepDSC employs a pipeline comprising six layers of Auto Encoder to reconstruct gene expression data. The hidden layer is extracted and subjected to convolution with the parameters of the Morgan fingerprint of the compounds. This convolved information is input for a DNN with four linear layers, predicting drug responses. The parameter settings for MOFGCN and DeepDSC were utilized from the original papers. We also include a DNN-based AutoML utilizing AutoKeras Jin et al. (2019). In addition, we utilize tree-based methods: LightGBM and Random Forest, tuning by FLAML Wang et al. (2021).

### 4.3 Comparative GNN Layer

Our study evaluates different Graph Neural Network (GNN) layers to determine their effectiveness. The layers compared are Message Passing Neural Networks (MPNN) Gilmer et al. (2017), Graph Convolutional Networks (GCN) Kipf and Welling (2016), Graph Attention Network (GAT) Veličković et al. (2017), Graph Attention Network V2 (GATv2) Brody et al. (2021), and Graph Transformer (GT) Shi et al. (2020). GAT introduces an attention mechanism into graph neural networks. It allows nodes to assign varying levels of importance to their neighbors dynamically. GATv2 is an advancement over GAT. It features a more expressive attention mechanism that recalculates attention coefficients in every forward pass of the model. The GT layer extends the Transformer Vaswani et al. (2017) architecture to graph-structured data. It leverages self-attention mechanisms specifically tailored for graphs.

### 4.4 Evaluation Metrics of Prediction Performance

To evaluate the prediction performance of our drGAT model, we employed four criteria: accuracy, precision, recall, and F1 scores. These metrics are defined as

(11) $$\begin{array}{c} \text{Accuracy}\;=\frac{TP\;+\;TN}{TP\;+\;TN\;+\;FP\;+\;FN},\\ \text{Recall}\;=\frac{TP}{TP\;+\;FN},\\ \text{Precision}\;=\frac{TP}{TP+FP},\\ \text{F1}\;\text{Score}\;=\frac{2\;\times \;\text{Precision}\;\times \;\text{Recall}}{\text{Precision}\;+\;\text{Recall}}, \end{array}$$

where TP, FP, TN, and FN correspondingly denote True Positive, False Positive, True Negative, and False Negative counts, respectively.

### 4.5 Hyperparameter Tuning

We utilized Optuna Akiba et al. (2019), a library that employs Bayesian Optimization to tune hyperparameters. Specifically, the number of epochs is 1500, the number of attention heads is 5, the number of hidden units is configured with 256 units for the first linear layer, 32 units for the second GNN layer, 128 units for the final GNN layer, the dropout ratio is 0.1, and the learning rate is 0.001.

### 4.6 Model Performance

In Table 1, we present the results of the drug response prediction. A comparison is made between four baseline methods and our model with several GNN layers. We train and fit the model 5 times and values show the average and standard deviation. Notably, GATv2 outperforms the other methods regarding accuracy, precision, and F1 score. GATv2 obtained a 0.791 average recall score while the best baseline method got only 0.724. We utilize GATv2 as the GNN layer in our model for further investigation. Furthermore, our model can provide interpretations based on attention coefficients that existing models cannot achieve.

## 5 Interpretation

### 5.1 Evaluation of Drug-Gene Associations in Scientific Literature

We conducted an analysis of drug-gene co-occurrences using Pubmed abstracts that examined the top 5 genes with the highest attention coefficients for each drug. We utilized the NCBI ESearch tool to retrieve abstracts that mention both the drug and gene of each relationship Coordinators (2012). Queries used the following format: https://eutils.ncbi.nlm.nih.gov/entrez/eutils/esearch.fcgi?db=pubmed&term="DRUG"&term="GENE".

**Figure 2:**
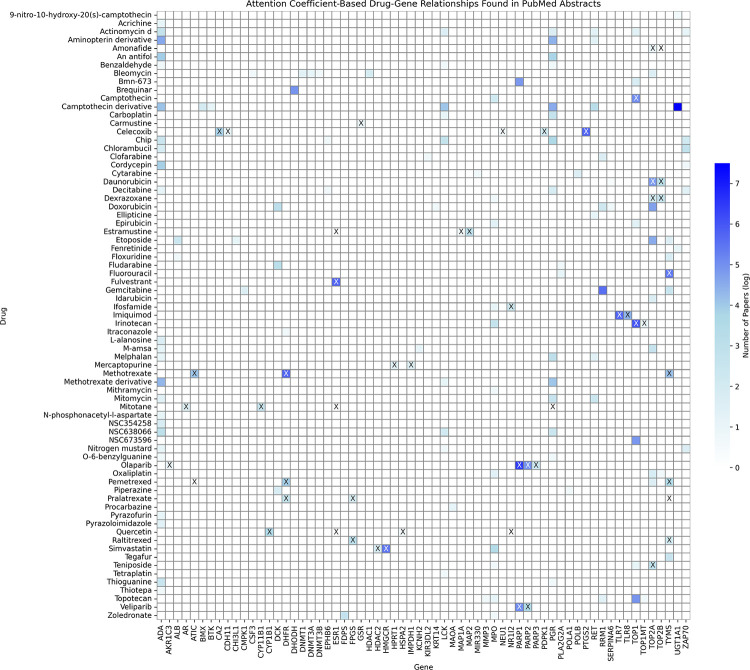
Drug-gene co-occurrences based on PubMed abstracts. The color represents the number of abstracts associated with a specific drug and gene pair by natural log scale. Several drugs have 5 or more co-occurrences because they have multiple NSCs.

Out of 1,345 drug-gene attention-based relationships, 165 of the drug-gene co-occurrences were found in the abstracts from 8843 articles. Figure 2 displays a heatmap illustrating the relationship between drugs and genes. The color corresponds to the number of related publications by log scale and the “X” shows the known drug-target interaction. Our model identified a wide array of genes, demonstrating its capacity to recognize more than just a few specific genes.

### 5.2 Evaluation of Predicted Drug-Cell Line Response by Comparison with GDSC

We systematically evaluated our results by comparing them with the Genomics of Drug Sensitivity in Cancer (GDSC) dataset Yang et al. (2012). The GDSC dataset is an extensive collection of drug response screening, which includes 978 cell lines and 542 drug compounds. The NCI60 subset we use here and GDSC overlap with 55 cell lines and 201 drugs. 44 lack drug-cell line response in NCI60, but exist in GDSC that are used for evaluation.

**Figure 3:**
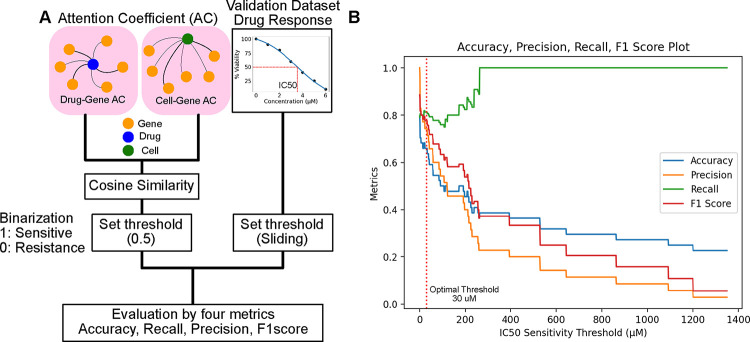
Evaluation using GDSC data. (A) Overview of calculation of 4 metrics. First, we obtained the attention coefficients between drug-gene and cell-gene. Then, calculate the cosine similarity. Next, we set the threshold to 0.5 to make it binary, 1 is sensitive and 0 is resistance. The GDSC observed drug response is given as IC50, and set some thresholds to make it binary (e.g., an IC50 > 20 *μM* is resistant). Then, calculate the 4 metrics: accuracy, recall, precision, and F1 score using the binary results. (B) Result visualization for different thresholds. We set the IC50 threshold from 1 to 1400 and describe the 4 metrics.

To assess these drug-cell line responses, we focused on their attention coefficients using our attention model, ensuring an accurate interpretation of their relationships. Our evaluation involved defining drug-cell sensitivity associations based on the attention coefficients of genes. We computed the cosine similarity between the attention directed towards genes from drugs and cell lines, selecting the cosine similarity due to its effectiveness in comparing sparse vectors. A cosine similarity value exceeding 0.5 was classified as positive (indicating sensitivity), whereas values below this threshold were deemed negative (resistant) (Figure 3A).

The GDSC dataset provides IC50 in micromolar (*μM*) units. IC50 is described as the "half-maximal inhibitory concentration," referring to the concentration of a substance (such as a drug or inhibitor) required to inhibit viability or a specific biological function by 50%. This enables us to determine drug sensitivity based on dosage. We calculated four key metrics: accuracy, precision, recall, and the F1 score, across various dose thresholds ranging from 1 to 1400 *μM* (Figure 3B). Figure 3B illustrates the impact of varying the sensitivity threshold value on the performance metrics of a classification model: accuracy, precision, recall, and F1 score. When the threshold is set low, the model achieves high accuracy and precision, identifying positive cases and minimizing false positives. However, a low threshold also leads to higher false negatives, as indicated by the lower recall. This scenario suggests that the model is conservative in predicting positive outcomes, leading to many positives being classified as negatives.

Conversely, setting a higher threshold improves recall, meaning the model is better at identifying all positive cases, including those that are harder to classify correctly. This comes at the expense of accuracy and precision as the model becomes more lenient, increasing the likelihood of false positives.

The balance between these metrics—accuracy, precision, and recall—highlights the trade-off in setting the threshold.

While each drug may be effective at different concentrations, lower IC50 concentrations tend to be more clinically relevant. The threshold where each metric (accuracy, precision, recall, and F1 score) achieves a balance for this analysis is around 30 *μM* with each metric approximately reaching a value of 80% (Figure 3B). This optimal threshold is one that balances performance across all four metrics, managing the trade-offs between identifying positive cases, minimizing false positives, and accurately classifying negative cases. Lower IC50 values can reflect the clinical relevance for compounds utilized in the analysis. The average IC50 value from the GDSC dataset is 100*μM*. The discrepancy between the average IC50 value and the IC50 threshold used for analysis may arise because of the binarization to distinguish between positive and negative cases.

Such insights are crucial for tuning classification models to achieve the desired balance between sensitivity (recall) and specificity (accuracy and precision), especially in applications where the cost of false positives and false negatives varies significantly.

### 5.3 Drug-Target Interactions Assessment from Attention Coefficients

**Figure 4:**
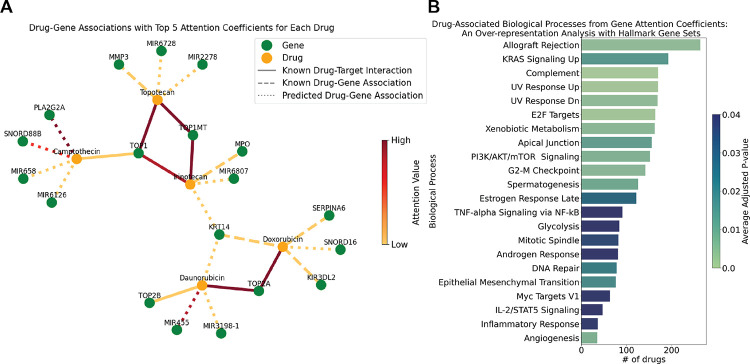
Drug-Gene Association Assessment. (A) Drug-gene association network based on attention coefficients. The network depicts relationships between drugs (orange nodes) and genes (green nodes) based on the top 5 attention coefficients from drGAT. For cases involving multiple NSCs, we only use data from a single NSC. Edges connecting drugs and genes are colored according to the attention coefficient value. Lines: known drug-target interactions (i.e., input training data; solid); known drug-gene association (relationships described in abstracts, but not included as model input, dashed); predicted drug-gene associations (dotted). (B) Drug-associated biological processes from gene attention coefficients. A bar chart representing the number of drugs linked to various biological processes, determined by gene attention scores and over-representation analysis of hallmark gene sets. The X-axis signifies the biological processes, while the Y-axis shows the count of drugs. The color coding of these bars corresponds to the average adjusted p-values.

The intricate network of drug-target interactions and quantifying their relationships from the model results through attention coefficients can aid our understanding of mechanisms of action. Figure 4 (A) presents a visualization of these drug-gene relationships, employing the drGAT model to highlight the interplay between chemotherapeutic drugs and their genetic targets.

Doxorubicin (NSC-759155 and NSC-123127) and daunorubicin (NSC-756717) are both anthracycline chemotherapeutic agents and are known as TOP2 inhibitors Chen et al. (2015). Results from drGAT retain this known drug-target association; TOP2A has the highest attention coefficient for both drugs. Doxorubicin and daunorubicin share an association with KRT14; KRT14 is a gene that is a member of the keratin 1 family. There is evidence that keratin expression is altered in response to doxorubicin treatment Liao et al. (2016) and are altered in doxorubicin-resistant cells (including specifically, KRT14) Greife et al. (2015). Additionally, there is interest in targeting keratins through peptide-drug conjugate featuring doxorubicin Saghaeidehkordi et al. (2021). Separately, SERPINA6, returned as a top 5 attention coefficient gene for doxorubicin, has been utilized as a resistance marker gene for doxorubicin De Ronde et al. (2013).

Topotecan (NSC-759263), Camptothecin (NSC-94600), and Irinotecan (NSC-759878) are topoisomerase inhibitors, specifically targeting the TOP1 cleavage complex Han et al. (2022), and the TOP1 drug-gene association appears in the drGAT results. MMP3 was returned as a drug-gene association for topotecan by drGAT. MMP3, and other matrix metalloproteinases (MMPs), have been analyzed as a part of a Phase I dosing study of intraventricular topotecan on children with neoplastic meningitis due to reports of correlation with leptomeningeal diseaseBlaney et al. (2013).

While not a comprehensive analysis, the above results show that our model retains high attention coefficients of known drug-target interactions fed as training data, and also, predicts several known drug-gene relationships that are of research interest. This suggests that our predicted drug-gene associations can be indicative of potential targets or drug-gene relationships appropriate for further study.

### 5.4 Over-Representation Analysis with Attention Coefficients

We worked to elucidate the functional roles of genes associated with the various drugs, leveraging the results provided by the attention coefficients derived from our model. We conducted an over-representation analysis (ORA) using the attention coefficients attributed to the genes associated with the drugs. Specifically, genes possessing attention values greater than 0 for each drug were taken as input for ORA. We conducted the ORA analysis using gseapy Fang et al. (2023) and the MSigDB Hallmark 2020 Liberzon et al. (2015) collection of 50 gene sets accessed via gseapy. We highlight the biological processes with p-values adjusted by Benjamini-Hochberg less than 0.05 in Fig. 4B, which depicts associations with different hallmark biological processes, with the cumulative count of drugs linked to each function.

Our study focused on drugs related to DNA damage. Consequently, our analysis reproduced processes represented in the Hallmark gene sets commonly associated with these drugs such as response to ultraviolet (UV) radiation, E2F targets (i.e., cell cycle-related targets of E2F transcription factors), xenobiotic metabolism, and DNA repair. This finding aligns with our initial hypothesis and these drugs’ known mechanisms of action. The E2F targets are crucial in cell cycle regulation and may represent how these drugs impact proliferation Hamidi et al. (2022). Similarly, the involvement of xenobiotic metabolism pathways highlights how these drugs are processed. Related, ABCB1 was a recurrent overalapping gene in the "KRAS Signaling Up" gene set; this drug efflux protein is responsible for reducing concentrations of toxic compounds, such as cancer medications, and it has been shown to be associated with the resistance to topoisomerase inhibition Pohl et al. (2011); Omori et al. (2022). The gene set "Allograft Rejection" was identified with the largest number of compounds. While not obvious from the name "Allograft Rejection" (i.e., transplant reject), this gene set contains many key cancer-related genes, including various kinases (e.g., EGFR, ITK, MAP4K1, LCK). Of these genes, one recurrent overlapping gene was EGFR for 80 of topoisomerase inhibitors (both TOP1 and TOP2; 130 topoisomerase inhibitors total); the various modes of interactions between topoisomerases and EGFR have been previously reported Chauhan et al. (2016). These results corroborate our expectations of the molecular mechanisms affecting response to these drugs.

## 6 Discussion

In this study, we successfully developed the drGAT model, utilizing Graph Attention Networks to derive attention coefficients for each node, including genes, drugs, and cells. This approach is capable of binary drug response prediction, as well as, facilitating model interpretation. Our model demonstrated superior performance in terms of accuracy, recall, and F1 score, outperforming competing models in drug response prediction. Additionally, the drGAT model is capable of predicting drug sensitivity in untested drug-cell line combinations based on attention coefficients, achieving over 80% accuracy. This capability is valuable for suggesting drug efficacy to untested samples (evaluated with untested samples in from the training data).

A key highlight of our research is the ability of our drGAT model to predict drug-gene relationships beyond those included in the training data as drug-target interactions (DTI). For drug-gene relationships that are not DTIs, our analysis found many relationships that have been previously researched using the attention coefficient-based approach. This capability allows our model to not only produce accurate response predictions that utilize known drug-target interaction information but, also, has the potential of identifying drug-gene relationships that could be of further interest in understanding drug mechanisms. Additionally, as part of this work, we analyzed the attention coefficients to understand the biological processes relevant to each drug. Our analysis returned several key processes related to cancer, as well as, processes with genes relevant to cancer and drug response.

One challenge in this study was the modest volume of high-confidence drug-target interaction data available. Although the NCI60 dataset includes 269 DNA damage-related medicines, only 100 drugs has drug-target interaction (for these drugs we included 571 interactions from DrugBank). This highlights the need for further collection of drug-gene relationships. Ongoing efforts by one of the present study’s authors and other colleagues are aimed at expanding such datasets. This expansion is actively utilizing advancements in information extraction from natural language processing as well as crowd-sourced curation efforts Wong et al. (2021); Bachman et al. (2023); Rodchenkov et al. (2020).

Overall, this work highlights how results from advanced machine-learning techniques can be paired with bioinformatic analyses to understand mechanisms of cellular response to treatment. We expect future work to broaden the approach, while providing more granularity to its interpretability. Currently, our model leverages gene expression data to generate cell similarity, but future work would incorporate additional omics data, including methylation, copy number variation, and mutation data as has been done by others Peng et al. (2021). We believe this enhancement will both boost model performance and interpretability.

### Implementation

We used PyTorch 0.13.1 Paszke et al. (2019), and PyTorch Geometric 2.2.0 Fey and Lenssen (2019). Our experiments were conducted on an Ubuntu server with an NVIDIA A40 GPU with 48 GB of memory.

## Figures and Tables

**Figure 1:**
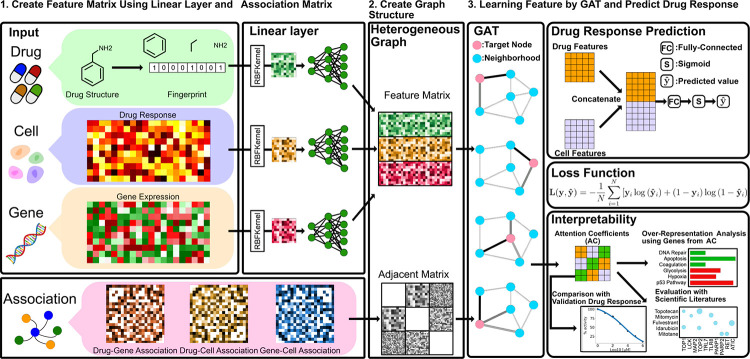
drGAT Overview. A heterogeneous graph is constructed using drug response, gene expression, drug structures, and association matrices, then used as GAT layer inputs. For prediction, the GAT layer output is concatenated to align with the drug response data. Following a fully connected layer, a sigmoid function generates predicted values $\overset{ˆ}{y}$, which are then inputs for the binary cross-entropy loss. To assess interpretability, attention coefficients are used with over-representation analysis and predicting untested sensitivities.

**Table 1:** Classification Performance. Model run 5 times with average performance metrics presented (variance indicated by ±).

	Method	Description	Data Structure	Interpretability	Accuracy	Precision	Recall	F1 score

**Baseline**	Deep DSC	AE	DF, GE	-	0.548 ± 0.000	0.516 ± 0.000	0.481 ± 0.000	0.498 ± 0.000
	MOFGCN	GNNs	DF, GE, MT, CNV	-	0.499 ± 0.001	0.487 ± 0.001	0.478 ± 0.002	0.482 ± 0.001
	Random Forest	Tree	DF, GE	Feature Importance	0.743 ± 0.003	0.720 ± 0.003	0.734 ± 0.006	0.727 ± 0.004
	LightGBM	Tree	DF, GE	Feature Importance	0.766 ± 0.000	0.791 ± 0.000	0.676 ± 0.000	0.729 ± 0.000
	AutoKeras	DNN	PCA+DF, PCA+GE	-	0.733 ± 0.007	0.709 ± 0.011	0.724 ± 0.015	0.716 ± 0.007

**drGAT**	MPNN	GNNs	DCG	-	0.620 ± 0.126	0.623 ± 0.129	**0.791** ± 0.182	0.664 ± 0.036
	GCN	GNNs	DCG	-	0.678 ± 0.036	0.720 ± 0.022	0.506 ± 0.119	0.587 ± 0.082
	GAT	GNNs	DCG	Attention	0.763 ± 0.018	0.730 ± 0.040	0.790 ± 0.045	0.756 ± 0.009
	GATv2	GNNs	DCG	Attention	**0.779** ± 0.002	**0.775** ± 0.013	0.741 ± 0.023	**0.757** ± 0.006
	Graph Transformer	GNNs	DCG	Attention	0.764 ± 0.01	0.739 ± 0.024	0.766 ± 0.025	0.752 ± 0.008

The best values are in bold. GNNs: Graph Neural Networks. AE: Autoencoder. Tree: Decision Tree. DNN: Deep Neural Networks. DCG: Drug-Cell-Gene Network. DF: Drug fingerprint. GE: Gene Expression. MT: Mutation. CNV: Copy Number Variation.
